# Crossing oceans

**DOI:** 10.7554/eLife.00477

**Published:** 2013-01-22

**Authors:** Eve Marder

**Affiliations:** Department of Biology and the Volen National Center for Complex Systems, Brandeis University, Waltham, United Statesmarder@brandeis.edu

**Keywords:** living science, careers in science, grad school, postdoc

## Abstract

**Eve Marder** explains why all scientists should spend time living and working in a foreign country.

As children we all read stories of explorers who crossed oceans and deserts in search of treasure, spices or the Fountain of Youth. It is hard to imagine that the search for treasure would be sufficient to drive individuals to embark on ocean voyages to unknown destinations in leaky wooden boats, but it did. It is hard to imagine people travelling for thousands of miles across difficult terrain to bring spices from the East to the courts of France and England, but they did. Whether or not these early explorers were of right mind, their experiences shaped their views of the world, and the tales they told expanded the universe for all those they encountered.

In 1975, under the auspices of a Helen Hay Whitney Postdoctoral Fellowship, I got on an airplane to start a postdoc in Paris. Almost everyone advised me not to go, saying I would never get a job in the United States if I left the country for a postdoc. In truth, I was leaving the United States at a time when I wasn't proud to be American, at the end of the war in Vietnam, and I didn't really know if I wanted a job. But I did know that there were beautiful papers in invertebrate neuropharmacology from the laboratories at the École Normale Supérieure (ENS) in Paris. So why wouldn't I go?

Of course, I hadn't the foggiest idea what my life in France would be. I had studied French in high school and college, and at age 18 could conjugate any French verb in any tense, and could read Rimbaud, Baudelaire and Proust. But I had never heard French spoken by the French (a high school French teacher with a Brooklyn accent didn't count). Nine years later, when I got off the plane at Charles De Gaulle airport, I couldn't understand a word. I spent my first year learning enough French to understand the conversation in the lab: almost everyone spoke English to me one-on-one, but the conversation immediately switched back to French as soon as there was a group of two. I found myself learning two languages simultaneously: French and enough biophysics to understand the experiments my new colleagues were doing.

**Figure 1:**
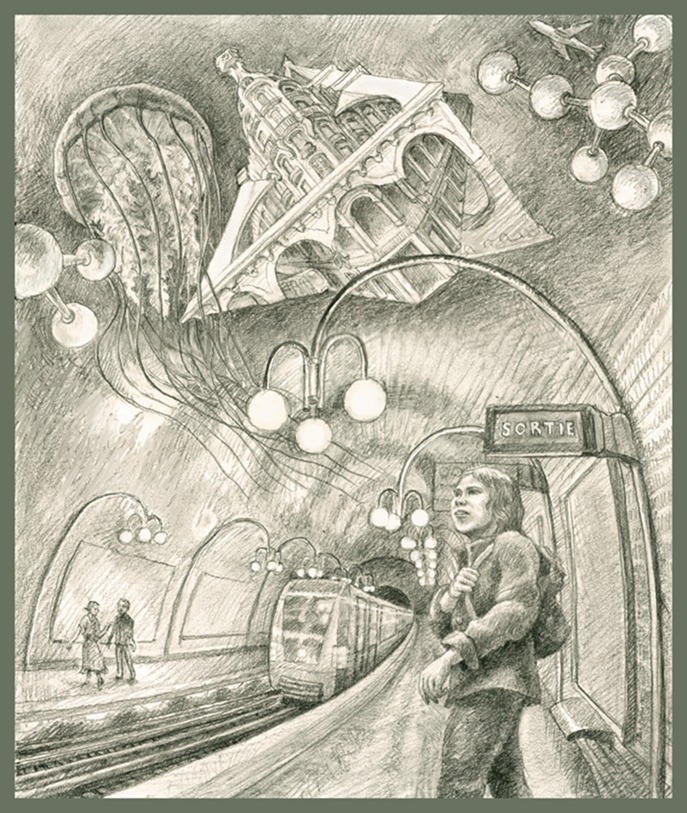
Spending three years as a postdoc in Paris in the 1970s was instrumental in Eve Marder being offered two faculty jobs in the United States at the end of her fellowship.

ILLUSTRATION: BEN MARDER

After a year I knew enough French to understand how deeply different the cultural attitudes to work, science, clothes, movies, food, politics, family and vacations were from mine. I discovered that people told me things that they didn't tell each other, because I was American and, therefore, I was exempt from ordinary rules of decorum. After two years I understood my new colleagues well enough to be able to predict what they were likely to do or say in most situations, although I was still shocked when a dentist chided me for not ironing my blue jeans. After three years I was comfortable in Paris, and ready to come home.

My years in Paris were wonderful years, filled with extraordinary people in an endlessly fascinating city I still love. But they were also very hard years, not so much because it took me a while before I was fluent enough in French to be part of a group and communicate, but because science is hard, and as a postdoc I didn't have the security and routine established by courses, qualifying exams, teaching duties and the peer group I had as a graduate student.

Moreover, having recently completed my PhD at the University of California at San Diego, which was then a brand-new institution with no tradition, it was disconcerting to be at one of the most elite institutions in France—an institution with a long history, and expectations and entitlements that were like nothing I had ever experienced. In San Diego I had been with people building a new institution with enthusiasm and blind confidence in the future they were creating: in Paris I had trouble understanding an education system that didn't allow people to start over later in life. (Most of those at the ENS had been stellar students throughout their education in a highly competitive school system; in the US, on the other hand, students had an opportunity redeem themselves if they suffered setbacks during their education.) Moreover, I had trouble understanding a scientific culture that gave life-time positions to people my age.

Because it is easy to communicate by email and Skype, it is also easy to underestimate the value of living and working in a foreign country.

Against all expectations, at the end of my time in Paris, I had two faculty job offers, both at excellent institutions. In retrospect I realized that I received those offers precisely because I had gone to France. The work I did there was not in itself spectacular, but somehow the fact that I had been adventuresome enough to leave home gave me the assurance to convince others that I was ready to be independent. In other words, I had dared to do what I was told not to do.

Science today has become a global enterprise and it is now possible to do fabulous science all over the world. And because it is easy to communicate by email and Skype, it is also easy to underestimate the value of living and working in a foreign country. No one I know who has lived abroad was unchanged by the experience (although today it is certainly easier to negotiate Paris without French than it was in 1975). But, getting on a plane in one country and getting off in a place where the sounds and sights are different, and the education system is different, brings perspectives that cannot be replaced by Skype.

Today American scientists are less likely to work abroad than are students from Asia, Europe or South America. Indeed, for many scientists from these regions, spending time abroad is necessary for them to be competitive when applying for a faculty position at home. The same is not true for an American scientist searching for a faculty position at an American university, although all of the Americans I know who spent time abroad feel immeasurably enriched by that time.

I urge every beginning scientist to get on an airplane to somewhere, to live and do experiments in a different country, even if only for a short visit. Scientists are supposed to be explorers! Go somewhere. Especially somewhere unexpected.

